# Therapists’ Experiences in Their Work With Sex Offenders and People With Pedophilia: A Literature Review

**DOI:** 10.5964/ejop.v14i2.1493

**Published:** 2018-06-19

**Authors:** Maria Hardeberg Bach, Carolin Demuth

**Affiliations:** a Department of Psychology, Southern University of Denmark, Odense, Denmark; b Department of Communication and Psychology, Aalborg University, Aalborg, Denmark; Department of Psychology, Webster University Geneva, Geneva, Switzerland

**Keywords:** sex-offenders, pedophilia, treatment providers, therapists, experience, personal impact, work-related stress, secondary traumatic stress, vicarious traumatization, compassion fatigue, burnout

## Abstract

This article presents a review of the literature that pertains to the experiences of therapists who work directly with child sex offenders and/or people with pedophilia. We draw together results from studies that attempted to identify how therapists experience such work and how they were personally impacted by it. Usually, such studies are embedded within one of the following theoretical frameworks: Secondary traumatic stress, compassion fatigue, vicarious traumatization and burnout. Most literature on the topic has therefore sought to determine to what extent and why, work-related stress responses may occur among these therapists. The aim of this paper is therefore to provide insight into this, arguably, important line of research, while evaluating the current knowledge as well as providing recommendations for future research efforts.

Since the 1980s, awareness and recognition of the problems related to the sexual abuse of children has grown drastically. While previously overlooked, it now commands the attention of professionals and lay communities alike as being a major societal issue (Taylor & Quayle, 2003). Although the prevalence of child sexual abuse has been difficult to determine, it was reported in a recent meta-analysis, which included 32 international studies, conducted between 2002 and 2009, that about 13% of girls and 6% of boys have experienced sexual abuse during their childhood (Barth, Bermetz, Heim, Trelle, & Tonia, 2013). As such, child sexual abuse is considered a prevalent, worldwide phenomenon. Furthermore, it is well known that such instances can be harmful to children. Studies on the long-term effects of child sexual abuse have, for example, consistently found that mental health problems are more prevalent among adults that experienced sexual abuse as children compared to those who did not (O’Leary, Coohey, & Easton, 2010).

In response to the public’s growing concern stemming from the aforementioned issues, criminal justice policies have expanded in an attempt to decrease the number of sexual offences against children. The psychological treatment of sex offenders and people with pedophilia is thought to be an essential part of such prevention strategies (Jahnke, Philipp, & Hoyer, 2015). In many countries, people who have been found guilty of committing a sexual offence against a child are therefore obliged by law to meet with a psychologist (Chudzik & Aschieri, 2013). In some countries, counseling options for people with pedophilia, who have not necessarily transgressed against a child, exist as well. The large German prevention project, “Dunkelfeld”, launched in 2004, is an example hereof (Cantor, 2014, p. 226).

At this point, it is clear that the therapeutic work with people who are sexually attracted to children is thought to be an important line of work that employs an increasing amount of professional personnel. However, not much is known about what it is like to work with this client group, as most of the emerging research on the topic has focused on “the victim” or “the perpetrator”, while less attention has been brought to the role of “the healer”. Furthermore, research that did focus on sex offender treatment tended to center on technique factors and its effectiveness, rather than the process of conducting such therapy (Friedrich & Leiper, 2006; Sandhu & Rose, 2012).

## Method

In the following, we draw together results from those studies that investigate how therapists personally experience the process of working with these clients. By gaining a clearer understanding of the challenges and rewards in this field, appropriate support can be given to treatment providers, ultimately strengthening the effect of psychological intervention for people who may commit sexual offences. To this end, we reviewed the existing literature on the topic in December of 2017 by consulting PsycINFO in a systematic manner (Boell & Cecez-Kecmanovic, 2010). We searched for the following terms: Pedophil* or *Sexual Abuse*, and different forms of the term therapist* (such as psychologist*). We only included publications available in English. Additional, specific publications were also identified using different strategies like “snow-balling” (Hart, 1998/2006) through several databases such as Google Scholar.

Publications that gave insight to one of the following questions where included, while excluding those that strictly focused on approaches to sex offender treatment and its effectiveness.

- How is work with sex offenders and people with pedophilia experienced by the treatment provider?
- What kind of impact can this type of work have on therapists’ psychological well-being?
- What explanatory factors have been provided in the literature thus far?

Our search yielded 18 empirical studies, relevant to these questions. An overview of these studies is provided in the Appendix (see Table A.1).

The terms “therapist”, “sex offender”, and “pedophile” were used as follows: “Therapists” refers to professional personnel from different backgrounds that provide treatment to the aforementioned clientele. While the term “child sexual abuse” is difficult to define (Haugaard, 2000), we understand the term as referring to any form of sexual contact between an adult and a minor. A “sex offender”, is therefore a perpetrator of a sexual offence against another person, in this review, almost exclusively those against children. The term “pedophilia”, on the other hand, refers to someone who is sexually attracted to children, regardless if that attraction is acted upon or not (Cantor, 2014).

## Results

### Reported Experiences and Effects Among Therapists Working With Sex Offenders and People With Pedophilia

#### Stress Responses to Interpersonally Demanding Work

The experience of traumatic stress has historically been associated with those individuals who have been the primary victims of traumatic events. More recently, however, there has been a growing realization that individuals who have been indirectly exposed to traumatic events may suffer as well (Ennis & Horne, 2003). Some occupations, such as that of the therapist, may entail continued exposure to other’s recollections of traumatic incidents. In turn, there may be a “cost to caring” (Hatcher & Noakes, 2010).

While no consistent term is currently available for these effects, four theoretical constructs have primarily been used to measure responses to (interpersonally demanding) work: *secondary traumatic stress* (Figley, 1995), *compassion fatigue* (Figley, 1995), *vicarious traumatization* (McCann & Pearlman, 1990) and *burnout* (Maslach, 1982). All four share significant similarities but have evolved from different theoretical frameworks, resulting in some variations (Jenkins, Mitchell, Baird, Whitfield, & Meyer, 2011). Whereas the concept of burnout is thought to capture stress reactions to any work environment, the three remaining constructs were originally developed to assess therapists working with trauma survivors specifically (Jenkins et al., 2011). Over time, however, all constructs have been used on a variety of populations (such as policemen, nurses, and so forth), but were not used to assess sex offender therapists, specifically, before the early 1990s (Crabtree, 2002).

#### Early Contributions

Farrenkopf (1992) conducted what is thought to be the first of these studies. Drawing on the secondary traumatic stress literature, he developed a questionnaire to assess the effects therapists faced from working with sex offenders. He found that more than half of the 24 therapists that participated in his study reported a negative shift in their perspective, as a result of working therapeutically with sex offenders. In more detail, participants experienced more anger and frustration, diminished hopes and expectations, and greater cynicism in their work. Other effects, such as suspicion and “emotional hardening”, occurred as well, not only with clients but also beyond the consulting room.

These results sparked an interest in this group of therapists. Soon after his study was published, a handful of studies, using methods similar to that in Farrenkopf’s study, were conducted. While the results of these studies were somewhat mixed (see Clarke and Roger, 2002 for a full review of these studies), Farrenkopf’s study remains one of the most frequently cited studies within this field, despite the small sample (*n* = 24). In fact, most literature that has dealt with sex offender therapists has portrayed this line of work as mentally, physically, and emotionally draining (Chassman, Kottler, & Madison, 2010; Scheela, 2001).

#### Contemporary Contributions

In 2004 Way, Van-Deusen, Martin, Applegate, and Jandle conducted a study, which supported the hypothesis that sex offender treatment providers may be negatively impacted by their work. They found that 52% of their sample of 347 therapists, working with sex offenders and/or victims, scored within the clinical range on the Impacts of Events scale. Nevertheless, the response rate for this study was low (23%), making it possible to questions the reliability of its results. Sabin-Farrell and Turpin (2003), for instance, highlight that those who feel negatively impacted by their work could be more likely to participate in such studies, as they may find them useful and relevant.

Based on their interpretive phenomenological analysis, Friedrich and Leiper (2006) also concluded that sex offender treatment providers experience a considerable amount of negative feelings, due to their work. In 2012, Sandhu, Rose, Rostill-Brookes, and Thrift also conducted a study using interpretive phenomenological analysis, in which similar results were obtained. It is, however, unclear to what extent positive experiences occurred as well. Although the authors stated that the participants were asked about positive aspects of work, no information hereof was included in their results.

Several authors have, however, reported either low levels of stress and/or a variety of positive experiences among sex offender therapists (Carmel & Friedlander, 2009; Elias & Haj-Yahia, 2016; Ennis & Horne, 2003; Hatcher & Noakes, 2010; Moore, 2016; Scheela, 2001; Shelby, Stoddart, & Taylor, 2001). In example, Carmel and Friedlander (2009) obtained low mean scores on stress-related measures, yet high levels of compassion satisfaction, indicating that the 106 therapists, who returned their questionnaire, generally enjoy their work. Scheela’s interview-study from 2001 generated similar results. According to her analysis via the constant comparison method, the participants felt like it was exciting to work in what was described as a new, challenging field. Interestingly, such findings are, however, seemingly overlooked in most of the literature in this field. Whether or not the majority of sex offender therapists, in fact, experience their work as mainly negative is therefore uncertain.

Finally, some studies of relevance to this issue have been inconclusive, reporting either mixed results or a lack of cut-off scores, making it difficult to evaluate the results (Adams, 2017; Crabtree, 2002; Steed & Bicknell, 2001; Thorpe, Righthand, & Kubik, 2001).

### Explaining Factors

While Farrenkopf (1992) did not hypothesize about what, specifically, leads to negative impact among therapists, other researchers have (Moulden & Firestone, 2007). Perhaps because of the dominant discourse within the literature (that sex offender therapy is strenuous work), most research efforts to date have sought to either locate stress-related symptoms within therapists or explain why they might occur. The following sections will examine these implicated factors, which can be organized into three explanatory categories: *client-focused*, *therapist-focused* and *organizational-focused* explanations (Moulden & Firestone, 2007).

#### Client-Focused Explanations

The *content* of work with sex offenders and people with pedophilia is considered by many to be central to what has been referred to as the “high incidence of reported psychological damage” among treatment providers (Clarke & Roger, 2002, p. 84). From this perspective, negative impact should be seen as a consequence of interacting with the, supposedly, unique characteristics of the client group. According to Pais (2002),

Unlike motivated clients who seek therapy voluntarily, sex offenders often try to deny, rationalize, and minimize their problems and are resistant to change. Working with this combination of factors can easily lead to what is commonly identified as ‘burnout’ in clinicians (p. 90).

In other words, these clients are perceived to be especially difficult to work with, due to such behavior. Categories like “sex offenders” may, however, be too heterogeneous to discuss as a single entity (Goldstein-Dwyer, 2014). These explanations could therefore lead to unjustified generalizations.

Furthermore, it seems to be a common belief that sex offenders are especially difficult to successfully rehabilitate, thereby, potentially, explaining negative responses among treatment providers. Defining “difficult”, “rehabilitation” and “success” in this context is, however, not an easy task (Chudzik & Aschieri, 2013). Although recidivism rates are not true offence rates, as many instances remain unreported, they could still provide insight into this issue. While recidivism rates vary be study (e.g. as a result of measurement variation), sex offenders have higher rates of general recidivism than sexual recidivism (Hanson & Morton-Bourgon, 2004). However, they have lower rates of general recidivism compared to non-sex offenders (Langan, Schmitt, & Durose, 2003). As such, sex offenders may be easier to successfully rehabilitate than non-sex offenders. In addition, treated sex offenders have lower rates of any recidivism compared to untreated sex offenders, indicating that treatment is, in fact, successful in many cases (Hanson, Bourgon, Helmus, & Hodgson, 2009). Consequently, difficulties in relation to rehabilitation, alone, cannot account for therapists’ distress.

That many argue that sex offender therapy is difficult because of factors within the client, as opposed to the therapist, is, however, unsurprising. According to all of the introduced theoretical constructs, therapists’ distress should be seen as a reaction to something *external* to the therapist (Clarke & Roger, 2007). What is surprising, on the other hand, is that none of the quantitative studies on this topic explicitly examined the inferred relationship between offender characteristics and therapists’ distress (Moulden & Firestone, 2007). It is therefore unclear if the assumed problem stems mainly from the client’s characteristics, exposure to, or knowledge about, sexual offences against children, a mixture of these, or something else entirely.

Studies using qualitative methodologies did, however, provide insight into this. Elias and Haj-Yahia (2016), who argued that existing research has merely sought to describe the consequences of work with sex offender, while neglecting how therapists perceive and cope with them, made the latter the aim of their research. According to one participant, negative impact was perceived to be a consequence of being confronted with their clients’ offences: “The emotions I carry with me are difficult. I think this intensive exposure to sexual deviance scars the psyche” (p. 10). This is in line with the argument put forth by Kottler and Markos (1997), claiming that,

One of the most unique challenges for therapists is working with pedophiles. Because of the reprehensible nature of the predatory behavior towards children, therapists need to be especially aware of how working with this type of offender will personally affect them” (p. 74).

#### Therapist-Focused Explanations

Many “moderating factors” within therapists have, however, been theorized. Yet, few studies incorporated these factors into their research. The role of treatment providers’ individual differences, in relation to the experience of work-related stress, has therefore not been studied widely or systematically (Sandhu & Rose, 2012). In the following section, we discuss research that includes such therapist-focused explanations.

##### Demographic Variables

Studies that included therapist-factors tended to focus on demographic variables, such as age or gender, which were correlated with test-scores on questionnaires (Crabtree, 2002; Steed & Bicknell, 2001). No clear patterns have emerged so far. Generally, such studies may provide basis for further research but their utility in terms of explaining individual differences appear to be low.

##### Coping Mechanisms

Some authors argue that the use of either positive or negative coping-mechanisms may mediate or moderate the effects of the work-content. According to Wallace, Lee, and Lee (2010), who administered a questionnaire to 232 “abuse-specific counselors”, the use of “active” coping strategies were linked to lower levels of burnout. Similarly, Thorpe, Righthand, and Kubik (2001), demonstrated that “positive coping mechanisms” were linked to improved work performance. These results were based on the administration of a questionnaire, which is thought to capture coping strategies and burnout potential, among 17 participants.

Moreover, Sandhu et al. (2012) suggested that staff used humor and various “emotional defenses” to deal with a range of negative emotions resulting from working with sex offenders. According to one participant, distress was managed by focusing on the social value of her efforts,

Actually hearing what they’re saying and how they’ve actually offended against a child or how they’ve managed to manipulate and rape a victim… is quite distressing and quite upsetting. But, you know you’re there to do a job and, and to help rehabilitate them so there are no more victims, that’s important” (pp. 300-301).

##### Therapists’ Personal Abuse Histories

Some research, which included therapist-factors, focused on therapists’ personal abuse histories. It has been hypothesized, that a large proportion of therapists who chose to work with sex offenders, were, in fact, abused as children, which in turn could account for the presumed high levels of work related stress among sex offender therapists (Moulden & Firestone, 2007).

Crabtree (2002) sought to test this hypothesis by correlating therapists’ reported childhood abuse incidences with test-scores on one of the abovementioned constructs. Results from this study, indicated that therapist with a personal abuse history showed more disruptions, compared to those who did not have a history of personal abuse.

Furthermore, 75% of the 252 sex offender therapists, who returned a questionnaire by Way et al. (2004), indicated that they had experienced some form of child abuse. Based on these results, it appears that a large proportion of sex offender therapists are, indeed, victims of abuse. However, no research to date has compared the rates of child abuse across specializations, making it difficult to draw any number of conclusions (Moulden & Firestone, 2007).

Interestingly, this topic also surfaced in a grounded theory study conducted by Chassman, Kottler, and Madison (2010). According to the therapists, who participated in the study and had experienced some form of abuse, such events had provided them with a particular lens, through which their clients were viewed. At times, this allowed them to see their clients with greater clarity. Sometimes, however, it blurred their vision. Based on these accounts, it may be the case that a personal history of abuse will affect how such clients are experienced – yet not necessarily in a negative direction.

While such hypotheses are interesting, as it is not inferred that dealing with sex offenders is necessarily traumatic in itself, there is currently not enough evidence to draw firm conclusions. More research, focused on therapists’ personal abuse history, is therefore warranted.

##### Negative Attitudes

Therapists’ conceptions about this client group may well influence how work with them is experienced. While numerous studies have focused on therapists’ attitudes towards this population (Adams & Betz, 1993; Elias & Haj-Yahia, 2016; Engle, McFalls, & Gallagher, 2007; Gore-Felton, Arnow, Koopman, Thoresen, & Spiegel, 1999; Fortney, Baker, & Levenson; 2009; Fuselier, Durham, & Wurtele, 2002; Harnett, 1997; Jahnke et al., 2015; Mellor & Deering, 2010; Reidy & Hochstadt, 1993), only one study focused directly on attitudes in relation to experience. Elias and Haj-Yahia (2016), who conducted an interview-study that was analyzed via grounded theory, illustrated that therapists’ perceptions about sex offenders personally affected them in their work. According to one participant, “A sex offender is a damaged person. He didn’t start out that way” (p. 1160). As such, the participant was able to empathize with her clients, driving therapy forward.

Previously, it was noted that an increasing amount of therapists work with sex offenders and people with pedophilia. Based on the following results, however, it would seem that, overall, few therapists chose this line of work, perhaps due to negative attitudes towards these types of clients. No more than 4,7% of the 86 German therapists that participated in a survey conducted by Stiels-Glenn (2010) indicated that they were willing to accept people with pedophilia for treatment. Furthermore, only 3,5% indicated that they would work with child sex offenders. Almost half of the participants that specified why they would not provide treatment for this group explained that such choices had been made due to negative feelings or experiences concerning these types of clients. The remaining half stated that they did not have enough knowledge about this client group to conduct treatment competently.

##### Lack of Support

Some therapists may decline such clients in order to prevent judgment from others in their community. Results from the previously introduced study by Scheela (2001), for example, suggested that sex offender therapists that spoke in opposition to the dominating, negative societal attitudes towards this population were viewed as the enemy as well. Some therapists, who chose to work with these populations, may therefore feel uncomfortable sharing what they do for a living with others, in fear of being misunderstood (Kottler & Markos, 1997). For these reasons, among others, collegial support among sex offender therapists is thought to be an important moderator of the effects of such work (Clarke & Roger, 2002).

##### Value Conflict

In order to conduct successful sex offender therapy many, potentially conflicting, demands require careful consideration. Since therapy with these populations is often court ordered, a frequently asked question is, for example, how to define clear boundaries between therapy and punishment (Chudzik & Aschieri, 2013). It can therefore be assumed that many therapists are faced with a dilemma when considering to whom they owe their loyalty – society or the offender?^i^

Another widely debated question concerns which approaches to sex offender treatment are the most effective and ethical. This relates, for instance, to what kind of stance therapists should take towards such clients. One school of treatment specialists contended that the first step in treatment should be to break denial and obtain disclosure by adopting a “confrontational approach” towards clients (Nori, 1992). In the 1990s this was the standard procedure in most sex offender treatment groups (Nori, 1992). Recommended therapeutic measures included various kinds of aversion therapy (Priest & Smith, 1992), for example, by pairing an arousing image with electrical shock (Quinsey, 1973). Drug therapy or “chemical castration”, which is still widely used in some countries but may entail intolerable side effects, is also an available tool (see Panesar, Allard, Pai, & Valachova (2011) for a detailed description hereof). Castration has also been used in extreme cases (Nori, 1992). Although these strategies have been widely criticized (Sandhu & Rose, 2012), discussions are still ongoing.

The abovementioned examples are included in this review because they highlight unique ethical dilemmas inherent in this line of work. While it is outside the scope of this review to discuss these questions in detail, we argue that such ambiguity may prove to be relevant in explaining potential negative effects from working with sex offenders, as such dilemmas may be strenuous to cope with. However, no researchers have, to our knowledge, focused on this potential relationship. Additional research that includes ethical aspects of these therapists’ work is therefore necessary.

#### Organizational-Focused Explanations

Some researchers investigated more general aspects of sex offender therapists’ work, such as their caseload, which was found to be the only significant predictor of burnout in a questionnaire study by Adams (2017). Similarly, Shelby, Stoddart, and Taylor (2001) identified the setting to be the only significant predictor of burnout in their study. In more detail, providing therapy for sex offenders in secure settings (such as prisons) was associated with greater stress among the 86 therapists, who participated in their study. This may be because secure facilities usually house sex offenders that are thought to be more dangerous. Consequentially, therapists that provide therapy for this group of clients may feel unsafe in doing their work. These findings suggested that the work setting could be the most powerful moderator, with regards to work-related stress among sex offender therapists.

As such, we should expect sex offender therapists to be exposed to general work-related stressors common across occupations. In other words, identified stress symptoms among sex offender may not (only) result from working with sex offenders. In some cases, then, negative impact may be better explained by drawing on theory from general work psychology.

## Discussion

In this review, we aimed at outlining the existing literature concerning sex offender therapists’ experiences and the effects of this line of work on these. These studies were, however, not without theoretical and methodological limitations, some of which will be assessed in more detail in this section.

### Sampling Bias?

Many research contributions within this field treated “sex offender therapists” as a homogenous group. However, therapists working with sex offenders come from a variety of educational backgrounds and provide counseling in various contexts. Searching for general experiences and impacts among these practitioners may therefore prove difficult.

Additionally, to our knowledge, research to date has been conducted exclusively on English-speaking therapists, with the exception on one study (Elias & Haj-Yahia, 2016). This is problematic as our conceptualizations about intergenerational sex are temporally and socially situated, which, in turn, will affect how therapists experience the process of working therapeutically with these clients, as well as what impacts we may expect hereof. Research should therefore be sensitive towards cultural issues (Angelides, 2005; De Block & Adriaens, 2013). Taking this into account, the population validity in these studies may be questioned. Nevertheless, this review only included publications available in English, meaning that publications in other languages, focusing on different populations, may have been overlooked.

Another sampling bias may be that most researchers drew their participants from organizations such as USA-based ATSA (Association for the Treatment of Sexual Abusers), which could further limit our possibilities to make any generalizations based on results from these studies.

### Theoretical Preconceptions?

Despite evidence to the contrary, working therapeutically with these clients was mostly portrayed as strenuous work in the discussed literature. One possible explanation for this apparent paradox may be that most researchers are aware of the results that were published by Farrenkopf (1992). Arguably, this awareness may have resulted in a heightened sensitivity towards negative experiences among therapists. Researchers in this field should therefore be aware of this potential confirmation bias.

Moreover, most researchers, including Farrenkopf (1992), based their research on one of the aforementioned theoretical constructs, in which therapists’ distress should be seen as a result to something external to the therapist, making the experience of distress among these therapists, to some extent, inevitable (Clarke & Roger, 2002). Currently, there is, however, little evidence to support this claim. It is therefore problematic that it is being inferred that therapists’ symptoms are, by nature, a consequence of their work.

Nevertheless, such research may be challenging to conduct for a number of reasons. As mentioned, categories like “sex offenders” and “pedophiles” include heterogeneous individuals, making it difficult to determine whether or not it would be meaningful to search for a unified ”nature”, which, in turn, could account for therapists’ “symptomology”. Similarly, “therapists” may not react uniformly to the demands of their work. Taken together, the time may therefore be ripe to explore other theoretical perspectives, in relation to sex offender therapists’ work experiences, than those associated with work-related stress.

### Methodological Preferences?

Because the majority of research in the field is based on questionnaire studies, these studies seem to fail to address important aspects of the experiences of treatment providers within this field. While the validity of several of these questionnaires have already been criticized, in relation to the lack of profession-specific questionnaires (Clarke & Roger, 2002), the following questions should also be considered in relation to these: What kind of knowledge can be gained from “measuring” stress-related symptoms and comparing different professions in relation to these? How should “stress” be operationalized in this context? What can these questionnaires tell us about causation? What does it mean to have “an experience” and can they be quantified in a meaningful way? And, finally, what can they teach us about the meaning of our experiences, individual differences, and contextual and dynamic aspects? In reference to the latter, longitudinal studies would be of value, as little is known about how “impact” may change over time, but do not currently exist.

In addition, no research to date included reference groups outside the field of sexual abuse, to our knowledge. Also, no norms (base-rates etc.) currently exist for clinicians (Moulden & Firestone, 2007). At this time, it is thus uncertain to what extent these effects are specific to work with sex offenders and people with pedophilia. Still, similar effects have been described in the literature pertaining to work with victims of sexual offences (Knight, 1997). Way et al. (2004), who compared counselors working with victims, against those working with offenders, found that levels of vicarious trauma did not differ between groups. According to the authors, “This is not to suggest that there is no relationship between vicarious trauma and client population. It is possible that the clinician groups in this study were too similar (…) For example, sexual offenders may also share details of their experience of sexual abuse victimization as a child” (p. 66).

Additionally, the results of these questionnaires, which are typically based on one of the aforementioned theoretical constructs, where usually correlated with, for example, demographic variables thereafter. While correlations may provide some useful insight into a phenomenon, claims of causality should be made with great caution (Coolican, 2009).

### Conclusions

We can conclude from the literature discussed in this review that some therapists who work with sex offenders are negatively affected by their work. There is, however, also sufficient evidence to determine that a considerable amount of these therapists find satisfaction in their work.

Overall, therapists working with sex offenders and people with pedophilia provide a vital contribution to our society. Enhancing our understanding of the challenges these therapists may face is therefore of great importance. Yet, in order to support treatment providers in their work, it should also be recognized that it is of equal importance to understand its rewards. As discussed in this review, most studies to date have, however, solely focused on determining the negative impacts of this line of work. Furthermore, a pursuit to identify, measure, and separate the negative impacts from the positive may be a questionable endeavor because such features, in fact, could be interrelated and coexist. It was, for instance, precisely the challenging nature of sex offender treatment that made it exciting to work with as well (Scheela, 2001).

Furthermore, we argue that “experience” must not be reduced to a concern with symptomology, as valuable aspects of human experience are left out. Instead, individual differences as well as dynamic and contextual features must be taken seriously, in order to achieve a holistic understanding of the issues in question. While not dismissing the value of questionnaire-based research, qualitative strategies may be better suited for this endeavor. Interpretive phenomenological analysis offers a promising avenue to this end, as a result of its consistent focus on the experiential content of consciousness (Smith, Flowers, & Larkin, 2009). Another line of research that might add important insights into the phenomenon, albeit from a different theoretical approach, is discursive psychology (Potter, 2012), which, amongst others, addresses issues of identity construction in talk (MacMartin & LeBaron, 2007).

## Appendix

**Table 1:** Description of Empirical Studies Directly Referring to the Experience of Working With Sex-Offenders and/or People With Pedophilia

Publication	Input or Evaluative Component	Methodological Paradigm	Results/Contributions to the topic
Adams (2017)	An exploration of 86 sex offender therapists’ level of burnout and their coping strategies. (Origin: USA/ATSA members).	Quantitative: Self-Report (Maslach Burnout Inventory and the COPE Inventory along with demographic questions).	Although, “the purpose of the study is to explore the level of burnout” (Adams, 2017, p. 53), there is no clear evaluation of the overall level of burnout among clinicians. Similarly, cut-off scores were not provided. Nevertheless, mean scores of EE = 21, DP = 8.5 and PA = 35, are not considered “high”, according to Maslach & Jackson (1986). Caseload size was found to be a significant predictor of the depersonalization aspect of burnout - DP (but not EE and PE). Coping, gender, and years of experience were not significant predictors of burnout.
Carmel & Friedlander (2009)	An exploration of 106 sex offender therapists’ level of work related stress in relation to their perception of the working alliance with clients. (Origin: USA/ATSA members).	Quantitative: Self-Report (Burnout, Compassion-Fatigue, Secondary Trauma and Compassion Satisfaction and demographic characteristics and perception of working alliance).	Analysis showed low mean scores on stress-related factors and high levels of compassion satisfaction, indicating that the participants enjoy their work.
Chassman, Kottler, & Madison (2010)	To explore 18 counselors’ experiences of adolescents with sexual behavior problems. (Origin: USA and Australia).	Qualitative: Interviews analyzed via grounded theory.	Positive and negative impacts and experiences were discussed. For example, the analysis revealed how the counselor’s own history of abuse could be seen as an advantage and a disadvantage in their work.
Crabtree (2002)	An exploration of the impact of previous trauma and gender on the experience of secondary trauma among 158 sex offender therapists. (Origin: USA/ATSA members).	Quantitative: Self-Report (The Traumatic Stress Institute Belief Scale, The Impact of Events Scale and demographic Information)	No significant results were obtained on the Impact of Events Scale. On the TSI Belief Scale, the respondents with histories of trauma did score significantly higher compared to those who did not, which, according to the author, means that therapists, who have suffered trauma, have greater disruptions in their cognitive schemas. Nevertheless, no cut-off scores were provided, making it difficult to evaluate the results on a group-level.
Elias & Haj-Yahia (2016)	Exploring the responses to work with sex offenders among 19 therapists (Origin: Israel).	Qualitative: Interviews analyzed via grounded theory.	Illustrated how therapists experience positive and negative impacts in their work and how these are dealt with.
Elias & Haj-Yahia (2017)	Exploring the attitudes related to sexual offending among 19 therapists (Origin: Israel).	Qualitative: Interviews analyzed via grounded theory.	Illustrated how attitudes towards sex offenders affected therapists in their work.
Ennis & Horne (2003)	An exploration of the experience of psychological distress among 59 sex offender therapists. (Origin: USA and Canada).	Quantitative: Self-Report (PTSD symptomology, family and peer support, work load and supervision hours).	As a group, the participants experienced low levels of distress. While support was significantly predictive of lower levels of distress, workload was not.
Farrenkopf (1992)	An investigation of the impact on 24 therapists from their work with sex offenders. (Origin: USA).	Quantitative: Self-Report (Impact of working with sex offenders, personal coping strategies and gender differences).	Based on an unspecified method of analysis the authors concluded that the respondents in their study experienced a negative shift in their perspective as a result of their work with sex offenders.
Friedrich & Leiper (2006)	An investigation of nine therapists’ responses to their work with a population of incestuous sexual abusers (Origin: UK).	Qualitative: Interviews analyzed via interpretive phenomenological analysis.	The authors concluded that treatment providers experience a considerable amount of negative feelings in their work. It is, however, noted that some therapists experienced positive feelings in relation to their clients, too, but this aspect was left out of the results.
Hatcher & Noakes (2010)	An exploratory study on compassion fatigue, burnout, vicarious traumatization and compassion satisfaction among 48 sex offender therapists. (Origin: Australia).	Mixed methods: Questionnaires (Professional Quality of Life Scale, Impact of Events Scale Revised, Quality of Work Life) and contained open-ended questions.	The analysis of the included questionnaires revealed low levels of vicarious trauma as well as low to moderate levels of compassion fatigue and burnout amongst the therapists. Furthermore, more than 85% of the participants reported moderate to high levels of compassion satisfaction, which indicates that many of the participants found their work satisfying.
Moore (2016)	The study aimed at uncovering countertransference issues among seven therapists working with male sex offenders. (Origin: USA/ATSA members).	Qualitative: Interviews analyzed via the consensual qualitative research approach.	The participants were provided questions that examined countertransference reactions with regard to positive feelings, negative feelings, and sexual feelings.
Steed & Bicknell (2001)	An exploration of the existence of work related stress in 67 therapists working with sex offenders. (Origin: Australia).	Quantitative: Self-Report (Fatigue-Self-Test for helpers, Impact of Events Scale Revised and years of experience).	None of the therapists were found to exhibit symptomology at a clinically significant level on the IES-R scale. From the results of the other scale, however, the authors concluded that, “as expected, working with perpetrators of sexual abuse was found to have a negative impact” (p. 5). In more detail, a mean score of 30.24 and SD of 13.6 were identified - within the potential range of 0-115. It is, however, difficult to evaluate these results, since no cut-off scores were provided. It is, furthermore, indicated that the test tends to err on the side of over-inclusion (Steed & Bicknell, 2001, p. 3). Moreover, the questions were changed from “past seven days” to “have you ever experienced…” (Steed & Bicknell, 2001, p. 3), also potentially resulting in over-inclusion. Finally, the hypothesis that work-experience would moderate burnout was not supported.
Sandhu, Rose, Rostill-Brookes, & Thrift (2012)	An exploration of the emotional challenges faced by staff (8) working on a sex offender treatment program. (Origin: UK).	Qualitative: Interviews analyzed via interpretive phenomenological analysis.	According to the analysis, the participants mainly experienced negative emotions in relation to their work.
Scheela (2001)	An investigation of 19 therapists, (Scheela’s own colleagues) about their experiences in their work with sex offenders. (Origin: USA).	Qualitative: Interviews were analyzed via the constant comparative method.	While not denying the challenges of this work, the participants generally described their experiences as positive and rewarding.
Shelby, Stoddart, & Taylor (2001)	To explore burnout 86 among sex offender treatment providers. (Origin: USA).	Quantitative: Self-Report (MBI/Burnout and informational questionnaire (gender etc.))	Overall, sex offender treatment providers did not obtain high scores on the MBI scales (as subscale scores were well under 27 and 13; at 19,6 and 8.21, respectively). Nevertheless, they scored higher than the mental health norm, yet lower than the social service worker norm. Moreover, sex offender treatment provider scored higher on “personal accomplishment” than the norm for both other groups - suggesting that sex offender treatment providers generally feel accomplished in their work. Furthermore, those working with incarcerated sex offenders obtained higher scores on burnout measures than outpatient treatment providers.
Thorpe, Righthand, & Kubik (2001)	Details the development of a new questionnaire and two empirical studies, which were made within this framework: First, an exploration of burnout-potential among 17 sex offender therapists and later a mixed group of professionals (*n* = 70) who provide sex offender evaluations. (Origin: USA).	Quantitative: Self-Report (Professional Impact Questionnaire).	The level of burnout differed between the groups of professionals. Clinicians showed intermediate scores (on the “emotional impact” category, which is thought to reflect potential for burnout). While the authors hypothesized that the scale can “measure burnout experienced by professionals” (Thorpe, Righthand & Kubik, 2001, p. 198), there is no clear evaluation of the overall level of burnout among clinicians. Similarly, indication of cut-off scores was not provided. Nevertheless, the use of positive coping strategies was linked to decreased burnout potential.
Wallace, Lee, & Lee (2010)	To investigate if coping strategies play a role in reducing burnout levels among 232 counselors in abuse-specific fields. It is, however, relevant to question the rationale behind this sampling strategy: How is it (dis)similar to work with substance abuse or child sex abuse? (Origin: USA/ATSA members).	Quantitative: Self-Report (Job Stress Scale, Brief COPE and Counselor Burnout Inventory).	The authors did not provide mean scores or cut-off scores, making it difficult to evaluate to what extent the participants should be considered negatively impacted by their work. Nevertheless, the results showed some mediation and moderation effects: Emotional coping strategies (such as humor) were linked to higher levels of burnout. Conversely, “active coping strategies” were linked to lower levels of burnout.
Way et al. (2004)	To explore and compare levels of vicarious trauma in a sample of 252 therapists who treat offenders and 95 therapists who treat victims. (Origin: USA/ATSA members).	Quantitative: Self-Report (Impacts of Events Scale and trauma history).	Groups did not differ significantly in overall VT-score. For the total IES, the mean score was in the moderate range, and 52% of the sample scored in the clinical range. Nevertheless, according to the authors, no cut-off scores are available for this questionnaire. Moreover, the response rate was 23%, which raises questions concerning reliability.
